# High-Fat Diet Induces Oxidative Stress and MPK2 and HSP83 Gene Expression in* Drosophila melanogaster*

**DOI:** 10.1155/2016/4018157

**Published:** 2016-08-07

**Authors:** Mariane Trindade de Paula, Márcia Rósula Poetini Silva, Stífani Machado Araujo, Vandreza Cardoso Bortolotto, Luana Barreto Meichtry, Ana Paula Pegoraro Zemolin, Gabriel L. Wallau, Cristiano Ricardo Jesse, Jeferson Luís Franco, Thaís Posser, Marina Prigol

**Affiliations:** ^1^Laboratório de Avaliações Farmacológicas e Toxicológicas Aplicadas às Moléculas Bioativas, Universidade Federal do Pampa (Unipampa), Campus Itaqui, 97650-000 Itaqui, RS, Brazil; ^2^Programa de Pós Graduação em Ciências Biológicas: Bioquímica Toxicológica, Departamento de Química, Centro de Ciências Naturais e Exatas, Universidade Federal de Santa Maria, 97105-900 Santa Maria, RS, Brazil; ^3^Centro Interdisciplinar de Pesquisa em Biotecnologia (CIP/BIOTEC), Universidade Federal do Pampa, Campus São Gabriel, 97300 000 São Gabriel, RS, Brazil

## Abstract

The consumption of a high-fat diet (HFD) causes alteration in normal metabolism affecting lifespan of flies; however molecular mechanism associated with this damage in flies is not well known. This study evaluates the effects of ingestion of a diet supplemented with 10% and 20% of coconut oil, which is rich in saturated fatty acids, on oxidative stress and cells stress signaling pathways. After exposure to the diet for seven days, cellular and mitochondrial viability, lipid peroxidation and antioxidant enzymes SOD and CAT activity, and mRNA expression of antioxidant enzymes HSP83 and MPK2 were analyzed. To confirm the damage effect of diet on flies, survival and lifespan were investigated. The results revealed that the HFD augmented the rate of lipid peroxidation and SOD and CAT activity and induced a higher expression of HSP83 and MPK2 mRNA. In parallel, levels of enzymes involved in lipid metabolism (ACSL1 and ACeCS1) were increased. Our data demonstrate that association among metabolic changes, oxidative stress, and protein signalization might be involved in shortening the lifespan of flies fed with a HFD.

## 1. Introduction

Obesity is a chronic multifactorial disease, result of positive energy balance, where food intake is greater than energy expenditure. This overweight predisposes the organism to a series of diseases such as cardiovascular problems, diabetes, and sleep apnea [1, 2]. Given that, excessive food intake is often directly linked to the consumption of foods rich in fat and the increase in the amount of fatty acids in the diet causes an imbalance in the metabolism [3].

A high-fat diet (HFD) causes damage at the cellular and molecular levels, and it triggers an oxidative stress process. Studies have demonstrated that this oxidative stress process generates different responses such as the activation of signaling pathways implicated in protecting cells against oxidative damage, such as heat shock proteins (HSP) and mitogen-activated protein kinase (MAPK) [3], peroxidation of lipids and modification of proteins [4, 5], and insulin resistance [6, 7]. Moreover, a HFD promotes an increased supply of triglycerides and fatty acids and consequently it results in an increase of fatty acids oxidation in order to produce energy.

Therefore, the study of biochemical mechanisms involved in the cellular responses to changes in the diet requires close attention to several metabolic pathways, given the complexity of the organism, since in cellular signaling pathways the interaction between genes and protein expression changes at the transcriptional level [8–11]. Acyl-CoA Synthetase and Acetyl-CoA Synthetase are enzymes present in the fatty acid metabolism, preserved in* D. melanogaster*, and they can be used as parameters to quantify the production of acetate, one of the main metabolites that play an important role in lifespan regulation [12].

In the last decade, drosophila has been a major model system for the study of obesity and metabolic syndrome combined with oxidative stress [13, 14]. Body fat in insects such as fruit fly* Drosophila melanogaster* plays a key role throughout its development, to meet the new physiological and energy needs [15]. Several phenotypic changes related to obesity in humans can be observed in the* Drosophila melanogaster* model, since flies exposed to a diet rich in fatty acids showed fat accumulation, cardiac dysfunction, increased levels of triglycerides, decreased levels of stress tolerance, and shortening of lifespan, reinforcing the idea of using drosophila as an excellent model [1, 16–18].

Studies focused on cellular stress facing the high consumption of lipids in drosophila are scarce. Thus, this work aimed to investigate the oxidative damage, antioxidant enzymes, and modulation of cells stress signaling pathway in response to a high lipid diet contributing to the knowledge about the cellular response to alteration of diet content in flies.

## 2. Materials and Methods

### 2.1. Materials and Fly Culture Condition

In order to perform the treatments, the following items were used: virgin coconut oil produced by Pró-Ervas®. Chemicals, including thiobarbituric acid (TBA), 2′,7′-dichlorofluorescein diacetate (DCF-DA), cocktail protease inhibitor, sodium orthovanadate, 4-(2-hydroxyethyl) piperazine-1-ethanesulfonic acid (HEPES), 5,5′-dithiobis(2-nitrobenzoic acid) (DTNB), acetylthiocholine iodide, ethylenediaminetetraacetic acid (EDTA), quercetin, N,N,N′,N′-tetramethylethylenediamine (TEMED), mannitol, 3-(4,5-dimethyl-2-thiazolyl)-2,5-diphenyl-2H-tetrazolium bromide (MTT), 7-hydroxy-3H-phenoxazin-3-one 10-oxide (Resazurin), and *β*-mercaptoethanol, were procured from Sigma-Aldrich Co., LLC, St. Louis, MO, USA. Fatty acid and Lipid Metabolism Antibody Sampler Kit and *β*-actin antibody were purchased from Cell Signaling Technology (Danvers, MA, USA). Triglycerides liquiform and glucose liquiform were obtained from Labtest (Lagoa Santa, MG, Brazil). The sodium dodecyl sulfate was procured from GE Healthcare Life Sciences (Little Chalfont, Bucks, ENG). Trizol Reagent and DNase I were obtained from Invitrogen (Grand Island, NY). iScript cDNA Synthesis Kit was from Bio-Rad (Laboratories, Montreal, Quebec). Tris(hydroxymethyl)aminomethane, hydrogen peroxide, TRITON X-100, and dimethyl sulfoxide were purchased from Synth (Diadema, SP, Brazil).

*Drosophila melanogaster* wild type (strain Harwich) was obtained from the National Species Stock Center, Bowling Green, Ohio, USA. The newly hatched flies were maintained for about 3 days in an incubator with controlled temperature of 25°C and 30–50% humidity under a light/dark cycle of 12 h fed on standard medium (1% yeast w/v, 2% w/v sucrose, 1% w/v milk powder, 1% agar w/v, and 0.08% v/w nipagin).

### 2.2. Experimental Diets: Regular Diet (RD) and High-Fat Diet (HFD)

All the flies were fed on a regular diet containing corn flour (76.59%), wheat germ (8.51%), sugar (7.23%), milk powder (7.23%), and salt (0.43%).* D. melanogaster* (both genders) aged from 1 to 3 days were divided in three groups of 30 flies each: (1) 0% coconut oil (regular diet, RD); (2) 10% coconut oil; (3) 20% coconut oil. The macronutrient compositions of the regular diet or coconut-supplemented diets are given in Table 1. The concentration of coconut oil used in this protocol is in accordance with Heinrichsen et al. [3].

### 2.3. Lifespan, Survival, and Body Weight

**Scheme 1:**
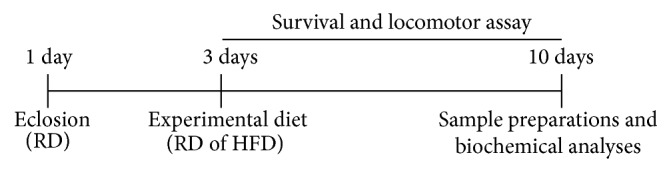


The flies were exposed to the treatments for 7 days; at the end of the treatments the flies were used for the different assays. The survival rate was evaluated by a daily count of the number of living flies until the end of the experimental period (Scheme 1). In addition, body weight was registered at the beginning and at the end of the treatment.

**Scheme 2:**
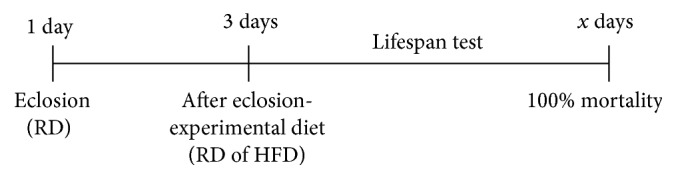


In order to test the lifespan, three days after the eclosion, the flies were maintained under different experimental diets which were changed every two days, until there were no more flies alive (Scheme 2).

### 2.4. Locomotor Assay: Negative Geotaxis

The locomotor performance of the flies fed with a RD and a HFD was investigated using the negative geotaxis test, according to Coulom and Birman [19], with minor modifications. Ten flies fed with a HFD and a RD were immobilized under light anesthesia with ice and they were placed separately in a vertical glass column (15 cm long and 1.5 cm in diameter). After 30 minutes of recovery from the anesthesia, the flies were gently tapped to the bottom of the column and the time they took to reach the height of 8 cm was recorded. The tests were repeated five times for each fly at on minute intervals. Results are presented as mean time ± SE (s) of three independent experiments.

### 2.5. Biochemical Assays

#### 2.5.1. Determination of DCF-DA Oxidation: Reactive Species Levels (RS)

For the quantification of reactive species generation (RS) a total of 20 flies were anesthetized on ice and homogenized in 1 mL 10 mM Tris-buffer, pH 7.4, after the end of the treatments. The homogenate was centrifuged at 1000 ×g for 5 minutes at 4°C and the supernatant was removed for assay quantification of 2′,7′-dichlorofluorescein diacetate (DCF-DA) oxidation, as a general index of oxidative stress according to the protocol of Pérez-Severiano et al. [20]. The fluorescence emission of DCF resulting from DCF-DA oxidation was monitored after one hour at an excitation wavelength of 485 nm and an emission wavelength of 530 nm in the spectrophotometer. The rate of DCF formation was calculated as a percentage of the fluorescence of the treatments in relation to the RD group.

#### 2.5.2. Determination of Lipid Peroxidation

In order to analyze lipid peroxidation, 20 flies were homogenized in 1 mL of HEPES buffer 20 mM, pH 7.0, and centrifuged at 1000 ×g for 10 minutes (4°C), after the end of the treatments. The supernatant was removed for assay, following the method of Ohkawa et al. [21], with minor modifications. Briefly, the supernatant was incubated in 0.45 M acetic acid/HCl buffer pH 3.4, 0.28% thiobarbituric acid, 1.2% SDS, at 95°C for 60 minutes, and the absorbance was then measured at 532 nm. Results represent the mean of three independent experiments (performed in duplicate). The TBARS values were normalized by protein concentration and are expressed as a percentage of TBARS production in relation to the RD group.

#### 2.5.3. Triglyceride, Glucose, and Protein Measurements

After seven days of exposing* D. melanogaster* to the diets, 20 whole flies were anesthetized, homogenized, and prepared, as described by Grönke et al. [22] for the analysis of total triglycerides and as described by Birse et al. [16] for the analysis of glucose. Both were measured by using the specific Labtest® kit. Protein concentrations of the homogenate were determined by the method of Bradford [23], using bovine serum albumin as the standard.

#### 2.5.4. Enzyme Assays

For the analysis of enzymes activity, 20 flies were homogenized in 1 mL of 10 mM Tris-buffer, pH 7.4, and then centrifuged at 1000 ×g for 10 minutes (4°C). An aliquot was taken for the analysis of acetylcholinesterase activity (AChE; EC 3.1.1.7) in a reaction mixture containing phosphate buffer (0.25 M, pH 8.0), 5,5-dithiobis 2-nitrobenzoic acid (5 mM), 50 *μ*L sample, and 25 *μ*L acetylthiocholine iodide (7.25 mM). The reaction was monitored for two minutes at 412 nm. Enzyme activity was expressed as nanomoles of substrate hydrolyzed per minute per milligram protein [24].

The remaining supernatant was centrifuged at 20000 ×g for 30 minutes (4°C) and it was used for the analysis of antioxidant enzymes. Catalase activity (CAT; EC 1.11.1.6) was determined according to Aebi [25] with minor modifications by Paula et al. [26]. The reaction mixture contained phosphate buffer (0.25 M/EDTA 2.5 mM, pH 7.0), H_2_O_2_ (10 mM), 0.012% Triton X 100, and 30 *μ*L sample. The decay in H_2_O_2_ was monitored during one minute at 240 nm and expressed as micromole of H_2_O_2_ decomposed/min/mg protein.

Superoxide dismutase activity (SOD, EC 1.15.1.1) was measured according to Kostyuk and Potapovich [27], with minor modifications by Franco et al. [28]. It was performed by monitoring the inhibition of quercetin autooxidation. The reaction mixture contained sodium phosphate buffer (0.025 M/EDTA 0.1 mM, pH 10), N,N,N,N-tetramethylethylenediamine (TEMED), and 10 *μ*L sample and it was started by adding 0.15% quercetin dissolved in dimethyl formamide. The reaction was monitored for two minutes at 406 nm, and it is expressed as the amount of protein required to inhibit 50% of quercetin autooxidation.

#### 2.5.5. Preparation of Mitochondrial Enriched Fractions and Metabolic Activity

A mitochondrial enriched fraction was prepared from the whole body of flies using the method of differential centrifugation [29]. Briefly, flies were homogenized in ice-cold Tris-sucrose buffer (0.25 M, pH 7.4) (60 mg fly tissue homogenized in 1000 *μ*L buffer) and centrifuged at 1000 ×g for five minutes (4°C). A fraction enriched with mitochondria was obtained by centrifuging the postnuclear supernatant at 10000 ×g for 10 minutes (4°C). The pellet was washed in mannitol-sucrose-HEPES buffer and resuspended in 200 *μ*L of suspension buffer.

A portion of 200 microliters of the mitochondrial enriched fraction (200 microliters) was incubated with 3-[4,5-dimethylthiazol-2-yl]-2,5-diphenyltetrazolium bromide MTT (x% solution) for 30 minutes at 37°C. After that, the samples were centrifuged at 10000 ×g for five minutes. The pellet was dissolved in dimethyl sulfoxide (DMSO), incubated for 30 minutes at 37°C, and the absorbance was measured at 540 nm. Results were expressed as percentage of the control.

#### 2.5.6. Dehydrogenases Activity of* Drosophila melanogaster*

An investigation of the dehydrogenases activity was carried out for the whole flies' homogenate and it was performed by using the CellTiter-Blue® cell viability assay kit [30]. The method is based on the ability of viable cells to reduce resazurin to resorufin, a fluorescent molecule. Whole flies were homogenized in 10 mM Tris-buffer, pH 7.4, and 100 *μ*L pipettedin, a 96-well plate, and an aliquot of CellTiter-Blue was added according to the instructions of the manufacturer. After 1 h, the fluorescence was recorded at _ex_579 nm and _em_584 nm.

### 2.6. Western Blotting

After the treatments, groups of 40 whole flies were mechanically homogenized at 4°C in 200 *μ*L of buffer (pH 7.0) containing 50 mM Tris, 1 mM EDTA, 20 mM Na_3_VO_4_, 100 mM sodium fluoride, and protease inhibitor cocktail. Then, the homogenate was centrifuged for 10 min at 1000 ×g at 4°C and the supernatant was collected. After protein determination according to Bradford [23], 4% SDS solution, ß-mercaptoethanol, and glycerol were added to samples to a final concentration of 100, 8, and 25%, respectively, and the samples were frozen for further analysis. The proteins were separated by SDS-PAGE using 10% gels and then electrotransferred to nitrocellulose membranes as previously described by Paula et al. [26]. Membranes were washed in Tris-buffered saline with Tween 100 mmol/L Tris-HCl, 0.9% NaCl, and 0.1% Tween-20, pH 7.5, and incubated overnight (4°C) with specific primary antibodies anti-Acetyl-Coenzyme A Synthetase (ACeCS 1) and anti-Acyl-Coenzyme Synthetase (ACSL 1) and anti-*β* actin. Subsequently, the membranes were washed in Tris-buffered saline with Tween and incubated for 1 h at 25°C with anti-rabbit Ig-secondary antibodies. Antibody binding was visualized using the ECL Western Blotting Substrate Kit (Promega). Band staining density was quantified using the Scion Image software (Scion Image for Windows). Results are expressed as optical density of ACSL1 or ACeCS1/optical density of respective ß-actin.

### 2.7. Quantitative Real-Time RT-PCR

A Trizol Reagent (Invitrogen, NY) based on RNA extraction was employed using 20 flies, according to the manufacturer's instruction. After quantification, the total RNA was treated with DNase I (Invitrogen, NY) and the cDNA was synthesized with MMLV RNAse H reverse transcriptase contained in the iScript cDNA Synthesis Kit (Bio-Rad), using both oligodT and random primers, following the manufacturer's protocol (Table 2). Quantitative real-time polymerase chain reaction was performed in 15 *μ*L reaction volumes containing 14 *μ*L of SYBR Select Master Mix 1x PCR Buffer plus 1 *μ*L of the sample containing 60 ng/*μ*L of cDNA in a 7500 Fast & 7500 Real-Time PCR System (Applied Biosystems, NY). The qPCR cycling protocol was the following: 50°C for two minutes and 95°C for two minutes, followed by 40 cycles of 15 seconds at 95°C, one minute at 60°C, and 30 seconds at 72°C. All samples were analyzed as technical and biological triplicates with a negative control. Threshold and baselines were manually determined using the StepOne Software v2.0 (Applied Biosystems, NY). RNA input normalization was performed with two genes (Tubulin and GAPDH), and the stability was evaluated and confirmed by geNorm [31]. The 2^−∆∆ct^ method [32] was used to calculate the relative expression.

### 2.8. Statistical Analysis

Lifespan measurement was determined by comparing the survival curves with a log-rank (Mantel-Cox) test. Other statistical analyses were performed using one-way ANOVA followed by Newman-Keuls* post hoc* test. Differences were considered statistically significant when *p* < 0.05. The Graph Pad Prism 5 Software was used for artwork creation.

## 3. Results

### 3.1. High-Fat Diet Reduces Lifespan and Survival Rate Causing Alteration in Body Weight, Triglycerides, and Glucose Levels

**Figure 1:**
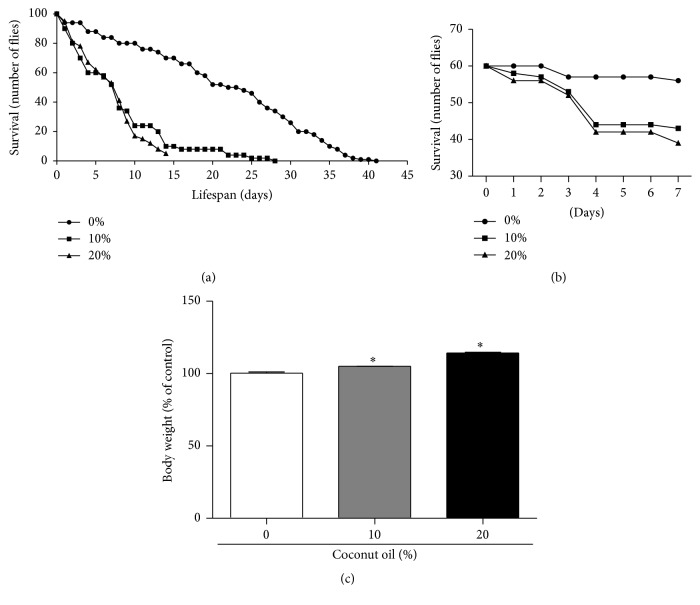
Lifespan and survival curves and body weight due to a high-fat diet. Wild type* Drosophila melanogaster* (strain Harwich: both sexes) were placed on regular diet (RD) and high-fat diet (HFD) under controlled conditions. (a) Lifespan: the flies were counted daily until there were no more flies alive. Coconut oil (10% and 20%) in the diet caused a significant decrease in lifespan. Four sets of twenty-five flies were used for each diet condition. (b) Survival curve in seven days: the number of live and dead flies was counted every 24 h during seven days of the treatment and exposure of diets. Coconut oil (10% and 20%) in the diet caused a significant decrease in survival rate. Three sets of twenty flies were used for each diet condition. (c) Body weight was evaluated at first and seventh days after the exposure to the different diets (data expressed in percentage of the 0% coconut oil group, RD). Three sets of twenty flies were used for each diet condition (mean ± standard deviation). ^*∗*^ *p* < 0.05 in relation to RD group.

According to Figure 1(a), the lifespan of flies receiving a RD was up to 41 days. However, when adding coconut oil at both concentrations tested (HFD: 10% to 20%), the maximum life span drops to 28 and 15 days, respectively. Furthermore, seven days of exposure of flies to a HFD (10% and 20% coconut oil) caused significant reduction on survival (28% and 35%, resp.) when compared with the RD group (*p* < 0.05) (Figure 1(b)). In addition, seven days of exposure to coconut oil in the diet (10% and 20%) caused a significant increase in the body weight of flies (5% and 14%, resp.) (Figure 1(c)).

**Figure 2:**
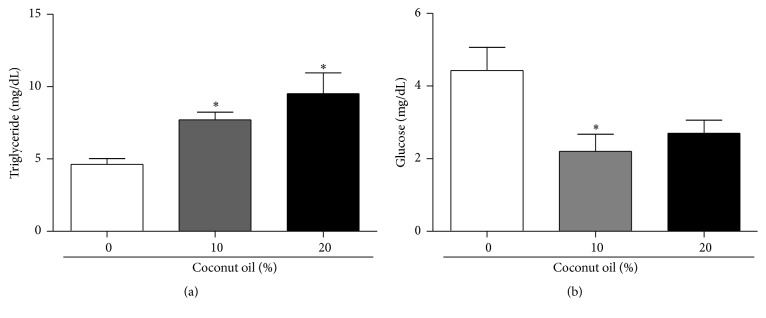
Diet enriched with coconut oil increased augmented triglyceride levels and decreased glucose levels in* D. melanogaster*. After seven days on the diets, three sets of twenty flies of each diet condition were used for triglyceride and glucose measurements. (a) The data expresses triglycerides levels in whole flies homogenate expressed in mg/dL. (b) Glucose levels in flies homogenate expressed in mg/dL. ^*∗*^ *p* < 0.05 in relation to the RD group.

The levels of triglycerides and glucose were measured in* D. melanogaster* exposed to a HFD. At the concentrations of 10% and 20% of coconut oil, the levels of triglycerides in flies increased 66% and 105%, respectively, when compared to the RD group (0% coconut oil) (Figure 2(a)). However, the HFD (10% and 20% coconut oil) caused a decrease in the glucose levels in 49% and 60%, respectively, when compared to the RD group (0% coconut oil) (Figure 2(b)).

### 3.2. Locomotor Performance and Acetylcholinesterase Activity

**Figure 3:**
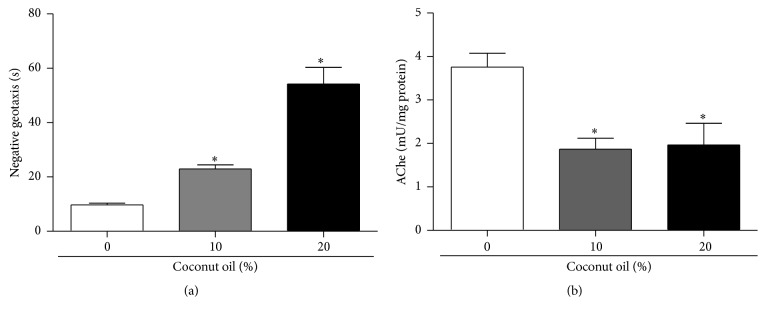
Effects of the exposure to a high-fat diet on locomotor performance and acetylcholinesterase (AChe) activity in* D. melanogaster*. (a) Locomotor ability of flies was analyzed by negative geotaxis after seven days of exposure to coconut oil enriched diet (0%, 10%, and 20%). Three sets of ten flies were used for each diet condition. Results are expressed as mean of time spent to reach 8 cm in a glass tube ± SE of three independent experiments. (b) The data shows the AChe activity in flies' homogenate expressed as mean (mU/mg protein) ± standard deviation. Three sets of twenty flies were analyzed for each diet condition. *∗* indicates a significant difference in the RD and the HFD (*p* < 0.05).

The exposure of flies to a HFD also had a significant deleterious impact on locomotor behavior. Flies fed for seven days with 10% and 20% coconut oil took two to five times longer to reach to the 8 cm measurement on the containers (Figure 3(a)). Thus, it is considered that the higher the concentration of coconut oil in the diet of drosophila, the lower their mobility capabilities in the negative geotaxis test.

Additionally, the activity of acetylcholinesterase (Ache) in* D. melanogaster* exposed to a HFD and coconut oil was also determined, which resulted in a decrease of AChe activity (Figure 3(b)).

### 3.3. Oxidative Stress Markers and Antioxidant Defenses

**Figure 4:**
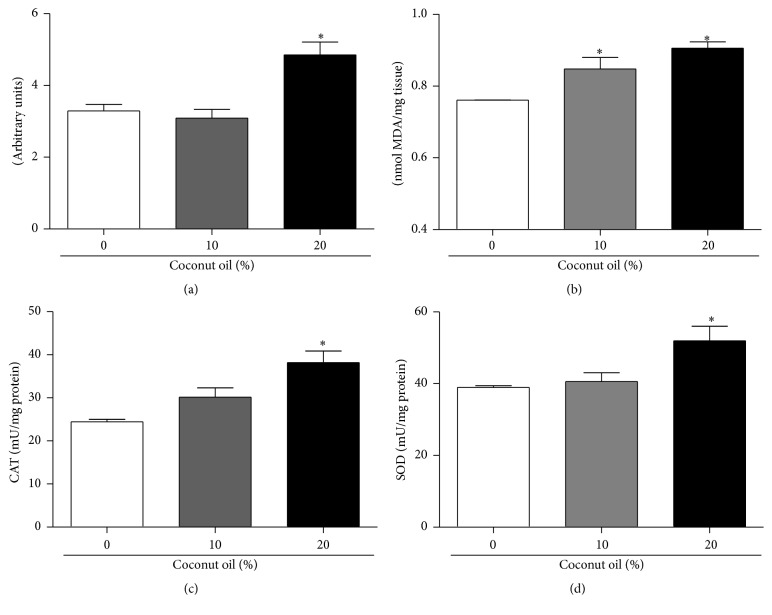
Effects on oxidative stress parameters in flies treated for seven days with a regular diet (RD) and a high-fat diet (HFD). After seven days on the diets, flies were homogenized and the supernatant was used for various analyses of stress markers and the activity of antioxidant enzymes. (a) Showing the DCF-DA intensity of fluorescence in total flies homogenate. Three sets of twenty flies were used for each diet condition. (b) End products of lipid peroxidation determined by TBARS assay in total flies homogenate, expressed in MDA nmoles/mg of protein. Three sets of twenty flies were analyzed for each diet condition. (c) Catalase (CAT) activity and (d) superoxide dismutase (SOD) activity in total flies homogenate. Three sets of twenty flies were analyzed for each diet condition. Data are expressed as a mean ± standard deviation in mU/mg protein. *∗* indicates a significant difference in relation to RD (*p* < 0.05).

Oxidative stress is directly linked to the accumulation of fat in mice and humans; however, the mechanism implicated in flies is not fully understood. In this study we quantify DCF-DA oxidation as a general indicator of oxidative stress and TBARS as an indicator of lipid peroxidation (Figures 4(a) and 4(b)). Flies fed with 20% coconut oil in the diet had a significant increase of 47% in the production of reactive species measured by the oxidation of DCF-DA in the RD group (Figure 4(a)). Regarding the levels of lipid peroxidation, both concentrations tested (10% and 20%) reported a significant increase of 11% and 19%, respectively, compared to the RD group (0% coconut oil) (Figure 4(b)).

The levels of the activity of the antioxidant enzymes CAT and SOD were determined (Figures 4(c) and 4(d)). After seven days of treatment, the flies that received 20% coconut oil concentration in the diet demonstrated a significant increase in the activity of both catalase (CAT) and superoxide dismutase (SOD) enzymes.

### 3.4. Metabolic Activity of Mitochondrial Enriched Fraction and Dehydrogenases Activity in Response to a HFD

**Figure 5:**
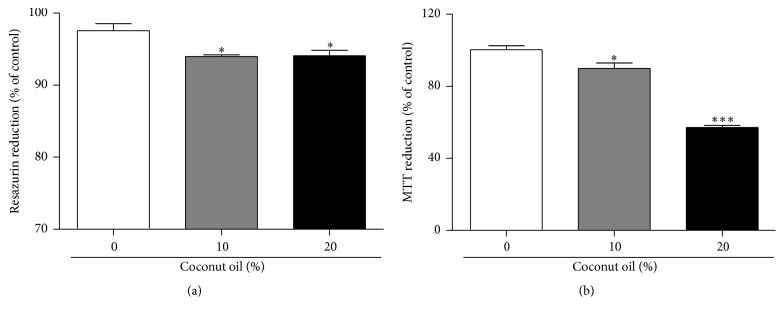
Effect of coconut oil on the metabolic activity and dehydrogenases activity in total homogenate of flies treated during seven days with a RD or a HFD. After seven days on the diets, flies were homogenized and centrifuged according to protocols and the samples were utilized for dehydrogenases activity (three sets of twenty flies were analyzed for each diet condition) by the resazurin reduction test in (a) and the metabolic activity (approximately 60 mg fly tissues were used for each diet condition) by MTT assay in (b). Both graphs express the results as a percentage (%) in relation to the RD group (mean ± standard deviation). ^*∗*^ *p* < 0.05 in relation to RD ^*∗∗∗*^ *p* < 0.0001 in relation RD.

Dehydrogenases activity was assessed by the resazurin reduction test and the metabolic activity was assessed by MTT assay. Both tests showed a significant drop in dehydrogenases and metabolic activity for all coconut oil concentrations tested (10% and 20%) (Figures 5(a) and 5(b)).

### 3.5. Coconut Oil Exposure in Diet by Seven Days Increases the Levels of ACeCS1 and ACSL1

**Figure 6:**
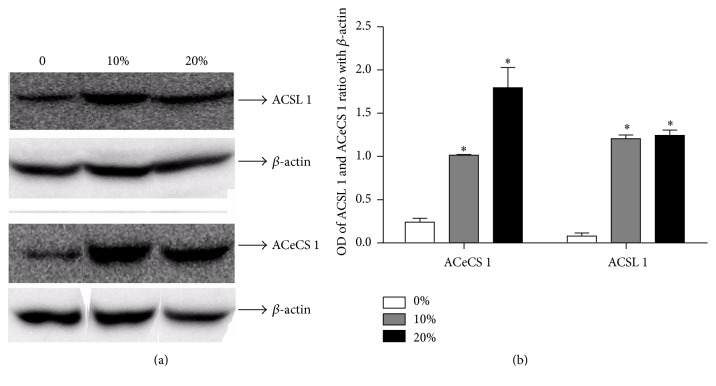
Expression levels of enzymes ACSL1 and ACeCS1 in response to the treatment of* D. melanogaster* with a regular diet (RD) and a high-fat diet (HFD) for seven days. After feeding the flies for seven days with different diets, they were homogenized and the proteins were separated by SDS-PAGE and transferred to nitrocellulose membrane. We quantified total content of proteins using specific antibodies. (a) The upper panel is a Western Blot showing expression levels of Acyl-CoA Synthetase (ACSL1) and Acetyl-CoA Synthetase (ACeCS1) with respective contents of *β*-actin. (b) The graphs are showing the ratio of OD from quantification of immunoreactive bands/*β*-actin and represent an average ± standard deviation. Three sets of forty flies were used for each diet condition. *∗* indicates a significant difference between the RD and the HFD (*p* < 0.05).

Acetyl-Coenzyme A Synthetase (ACeCS 1) and Acyl-Coenzyme Synthetase (ACSL 1) were investigated in flies exposed to coconut oil (10% and 20%) in the diet during seven days. There was a significant increase in ACeCS1 content in the groups that have consumed 10% and 20% of coconut oil in diet, respectively, when comparing to control group (Figure 6(b)). ACSL1 content was increased in both concentrations tested comparing to control (Figure 6(b)). Data expresses a ratio of optical density of the bands in relation to *β*-actin.

### 3.6. Quantitative Real-Time PCR (QRT-PCR) Analysis of HSP83, MPK2, SOD, and CAT mRNA in Flies

**Figure 7:**
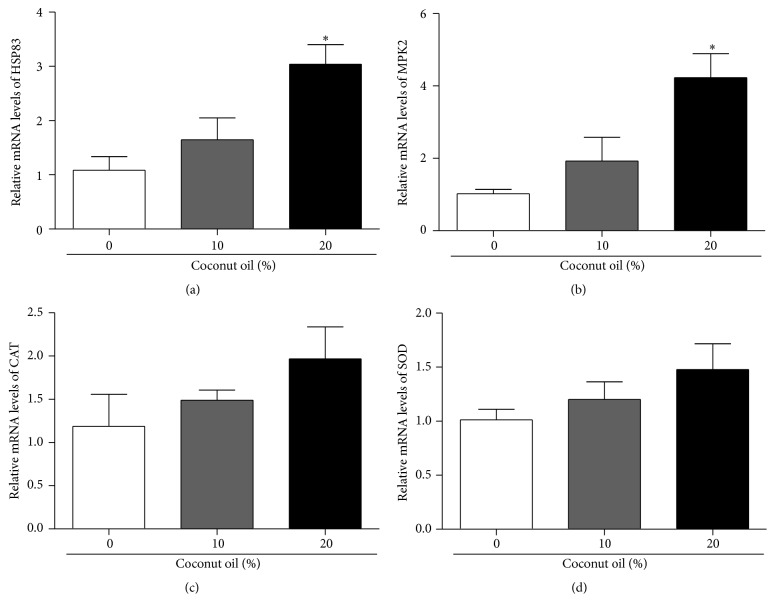
Quantitative real-time PCR (qRT-PCR) analysis of Hsp83, MPK-2, CAT, and SOD mRNA in flies exposed to high-fat diet. Flies were exposed for seven days to 0%, 10%, and 20% of coconut oil in their food. qRT-PCR was used to quantify levels of mRNA of each diet after exposure. The data were normalized against TUBULIN transcript levels and each bar represents the mean ± SEM. Three sets of twenty flies were used for each diet condition. *∗* indicates a significant effect of a high-fat diet in comparison with a RD (*p* < 0.05).

Flies were exposed to 0%, 10%, and 20% coconut oil in their food for seven days. We used qRT-PCR to quantify levels of mRNA, relative to the respective RD groups, after exposure. The data were normalized against TUBULIN transcript levels. The concentration of 20% coconut oil causes significant increase in HSP83 and MPK2 expression of mRNA levels (Figures 7(a) and 7(b), resp.). Although the statistical tests did not reveal significant difference, SOD and CAT mRNA levels seem to be increased in relation to control group, mainly in higher concentration of oil (Figures 7(c) and 7(d), resp.).

## 4. Discussion

The results of this study indicate that the coconut oil, when added to the diet of the* Drosophila melanogaster*, causes several changes in its metabolism as observed in studies based in mammal models [32]. Coconut oil in both concentrations tested (10% and 20%) is one of the causes of a significant increase in body weight, triglyceride levels, and a decrease in glucose levels. Additionally, an increase in the enzymes present in the metabolism of fatty acids, such as Acetyl-CoA Synthetase (ACeCS1) and Acyl-CoA Synthetase (ACSL1), and an induction of oxidative stress were observed. Possible serving as an adaptive response to this, there was an increase in the activity of antioxidant enzymes SOD and CAT and augmented expression levels of mRNA of Hsp83 and MPK2. Furthermore, the locomotor performance was impaired and the activity of acetylcholine esterase was reduced. Besides, cellular and mitochondrial viability was decreased with a significant reduction in the lifespan of treated flies.

The addition of coconut oil concentrations in the diet (10% and 20%) increases the body weight and triglycerides levels. Considering the diet being tested, which is high in saturated fat, it offers to flies larger concentrations of fat and, consequently, the increase of body weight is attributed to the increase of fat amount. It has been demonstrated, corroborating with our study, that a high-fat diet induces several consequences on lifespan, stress tolerance, and others in the* Drosophila melanogaster* model [3, 17]. In our study, we observed the decrease in glucose levels in flies fed with 10% and 20% coconut oil concentrations in their diet; however, our result is different from those observed by others [3, 17, 33]. According to Heinrichsen et al. [3] flies that received a high-fat diet (combination of yeast, corn starch, molasses, and 20% coconut oil concentration) demonstrated an increase in glucose levels. We suppose that the increase in the availability of triglycerides and fatty acids alters the use of glucose and its substrates on the oxidative mitochondrial metabolism. In fact, high-fat diets are known to influence the glucose metabolism including the increase in lactate levels in obese human subjects and the increase of lactate and pyruvate in mice that received a HFD [6, 7].

Many of the metabolic regulatory pathways are deranged in models using high-fat diets. A HFD promotes an increased supply of triglycerides and fatty acids and consequently an increase of fatty acids oxidation as a way to get energy. Our work demonstrates for the first time an association between a HFD and the increase on Acyl-CoA Synthetase and Acetyl-CoA Synthetase phosphorylation, enzymes that are present in the fatty acid metabolism, in* Drosophila melanogaster*.

The significant increase in Acyl-CoA Synthetase in the flies is directly connected to Acetyl-CoA by the oxidation of fatty acids for adenosine triphosphate (ATP) production in the Krebs cycle. Therefore, we suggest that the HFD is being used to produce energy since the glucose levels are not enough. However, we know that the excess of Acetyl-CoA by oxidation of fatty acids can generate ketone bodies and acetate. Some studies demonstrate that acetate levels rise when fatty acid oxidation rises [34]. Acetate freely diffuses to most organs where it is utilized by Acetyl-CoA Synthetase to generate Acetyl-CoA. Then, Acetyl-CoA can be oxidized by peripheral tissues as a source of energy [34]. The increase in Acyl-CoA Synthetase and Acetyl-CoA Synthetase in our study is related to the increase in this metabolic pathway caused by a HFD in the mitochondria.

Given that a HFD accelerates the lipid metabolism route and increases the Acetyl-CoA Synthetase enzyme, we can suppose an increase in the amount of acetate available in the organism of the flies fed with coconut oil and, in part, this is one of the causes of the reduction in the lifespan, as presented in this study, since many authors report than acetate metabolism plays a critical and important role in aging, because it is regulated by NAD^+^ dependent protein deacetylases (sirtuins) that have central roles in energy homeostasis and aging.

Mitochondria are cytoplasmic organelles whose main function is the production of most of the phosphate compounds needed for energy balance of the cell [35]. Besides, the mitochondrial dysfunction can accumulate oxidative damage, increase the RS generation, and consequently decrease ATP production and cell viability. The mitochondrial activity in excess by an increase of enzymes ACSL1 and ACeCS1, observed in our study, can be directly related to the increase in the RS production and, consequently, oxidative stress caused by a HFD in our model. In fact, in our study we observed an increase in RS production in the flies fed with 20% coconut oil concentration in the diet. Excessive consumption of fats, as in the cases of obesity, increases mitochondrial oxidative work load, which causes an increase in mitochondrial RS production by the electron transport chain and, consequently, the oxidative stress situation [36]. We believe that the increased oxidative stress is directly related to the change in the diet, providing flies with increased intake of saturated fats present in the commercial coconut oil.

Moreover, the oxidative stress by a HFD also caused an increase in lipid peroxidation. Furthermore, studies described that this high-fat diet model based on coconut oil concentrations is characterized as a genetic model to study obesity in* D. melanogaster* [9, 16, 37]. Increased oxidative stress in accumulated fat is an important pathogenic mechanism of metabolic syndromes associated with obesity, because oxidative damage may favor cell damage processes and inflammatory processes [38]. As a compensatory response to the damage, there is an increase in the activity of the antioxidant enzymes CAT and SOD and, in addition to this, there is a tendency to increase the expression of these enzymes. We believe that this increase in the activity of antioxidant enzymes and expression may represent a compensatory response to oxidative insults, since chronic exposure to coconut oil remained for seven days.

Obesity caused by lipid accumulation in adipose tissue is responsible for triggering cellular stress, a process of chronic inflammation characterized by abnormal production of cytokines by adipose tissue [39]. Once in a state of stress, the major organelles that suffer the consequences of this stress are the mitochondria and the endoplasmic reticulum (ER) [40]. In this study, a decrease in the metabolic activity has been demonstrated confirming the presence of a stress state which may result from apoptotic processes that produce a decrease in dehydrogenases activity caused by coconut oil in the diet of* D. melanogaster*. Besides, in increased nutrition conditions, the adipocyte breaks its ER which begins to generate malformed proteins. In response to this damage, the adipocyte ER begins to generate responses as the formation and increased expression of heat shock proteins and mitogen-activated protein kinases (MAPKs) [41].

In invertebrates, MAPKs and molecular chaperones such as the heat shock family of stress proteins (HSPs) participate in a range of cellular processes, including the development of normal cells, regulating the immune response and cytoprotection [42, 43]. However, there are no studies regarding the effects caused by a HFD with coconut oil in flies on gene expression of MAPK mRNA levels and HSP83 mRNA levels, a heat shock protein. Flies on a HFD with 10% and 20% coconut oil concentrations also led to the cellular response to damage such as increased expression of heat shock protein HSP83 and increased expression of MPK2, which is the* D. melanogaster* protein homologous to the P38 (MAPKs) in mammals.

In our experimental protocol, the exposure of* D. melanogaster* to coconut oil concentrations (10% and 20%) revealed a decrease in locomotor capacity by changes in geotaxis negative, a commonly used behavior addressed to assess neurolocomotor function in* Drosophila melanogaster* [44]. Moreover, a decrease in the activity of the enzyme acetylcholinesterase was also observed. Fournier et al. [45] reported that changes in the enzyme AChe, present in the central nervous system of drosophila, can affect the sensitivity of these insects and their locomotor ability as demonstrated in compounds with insecticidal effects. Furthermore, a reduction in the growth of skeletal muscles which can be directly related to the decrease in protein levels and locomotor capacity has been reported in studies of mice that received a high-fat diet in comparison to the study model reported in this work [3]. In addition to this, we presuppose that because the flies treated with coconut oil showed a significant decrease in glucose levels, the degradation of fatty acids by lipolysis is a compensatory role.

In summary, our study revealed for the first time that flies fed for seven days with a high-fat diet by coconut oil addition (10% and 20%) have a reduced locomotor performance, an increase in Acyl-CoA Synthetase and Acetyl-CoA Synthetase, and, consequently, an increase in the production of reactive species and thiobarbituric acid reactive substances, an increase of HSP83 and MPK2 expression, which generates an oxidative stress situation. Furthermore, the association among metabolic changes, oxidative stress, and protein signalization might be involved in shortening the lifespan of flies fed with a HFD.

## Figures and Tables

**Table 1:** Composition of regular diet (RD) and high-fat diets (HFD).

	RD	HFD 10%	HFD 20%
Energy (kcal/g)	4.039	4.49	4.96
Carbohydrate (weight, %)	89.26	80.13	72.55
Protein (weight, %)	8.66	7.77	7.04
Total fat (weight, %)	2.07	12.08	20.40

Total saturated fat (weight, %)	1.08	10.00	17.71
6:0	0	0.03	0.07
8:0	0	0.05	0.10
10:0	0	0.49	0.94
12:0	0.03	0.61	1.13
14:0	0.13	2.02	3.6
16:0	0.61	1.71	2.47
18:0	0.20	0.61	0.91
20:0	0.01	0.01	0.01
24:0	0.001	0.002	0.002

Total monounsaturated fatty acids (weight, %)	0.60	1.35	1.81
14:1	0.007	0.01	0.01
16:1	0.02	0.04	0.06
18:1	0.55	1.30	1.78
20:1	0.002	0.003	0.003

Total polyunsaturated fatty acids (weight, %)	0.38	0.72	0.88
18:2 n-6	0.40	0.63	0.69
18:3 n-6	0.01	0.02	0.03
18:1t	0.04	0.06	0.08
18:2t	0.005	0.007	0.009

HFD 10% and HFD 20%: high-fat diets were performed with a coconut oil addiction.

**Table 2:** Genes tested by quantitative real-time RT-PCR analysis and used forward and reverse primers.

Genes	Primers sequences
Tubulin	LEFT 5′-ACCAATGCAAGAAAGCCTTG 3′
	RIGHT 5′-ATCCCCAACAACGTGAAGAC 3′

Catalase	LEFT 5′-ACCAGGGCATCAAGAATCTG 3′
	RIGHT 5′-AACTTCTTGGCCTGCTCGTA 3′

Superoxide dismutase	LEFT 5′-GGAGTCGGTGATGTTGACCT 3′
	RIGHT 5′-GTTCGGTGACAACACCAATG 3′

HSP83	LEFT 5′-CAAATCCCTGACCAACGACT 3′
	RIGHT 5′-CGCACGTACAGCTTGATGTT 3′

MPK2	LEFT 5′-GGCCACATAGCCTGTCATCT 3′
	RIGHT 5′-ACCAGATACTCCGTGGCTTG 3′
